# Systems analysis of the transcriptional response of human ileocecal epithelial cells to *Clostridium difficile *toxins and effects on cell cycle control

**DOI:** 10.1186/1752-0509-6-2

**Published:** 2012-01-06

**Authors:** Kevin M D'Auria, Gina M Donato, Mary C Gray, Glynis L Kolling, Cirle A Warren, Lauren M Cave, Michael D Solga, Joanne A Lannigan, Jason A Papin, Erik L Hewlett

**Affiliations:** 1 Department of Biomedical Engineering, University of Virginia, Charlottesville, Virginia, 22908, USA; 2 Division of Infectious Diseases and International Health, Department of Medicine, University of Virginia, Charlottesville, Virginia, 22908, USA; 3 Department of Microbiology, University of Virginia, Charlottesville, Virginia, 22908, USA

**Keywords:** *Clostridium difficile*, Toxin A, Toxin B, gene expression, epithelial cell, cell-cycle

## Abstract

**Background:**

Toxins A and B (TcdA and TcdB) are *Clostridium difficile*'s principal virulence factors, yet the pathways by which they lead to inflammation and severe diarrhea remain unclear. Also, the relative role of either toxin during infection and the differences in their effects across cell lines is still poorly understood. To better understand their effects in a susceptible cell line, we analyzed the transciptome-wide gene expression response of human ileocecal epithelial cells (HCT-8) after 2, 6, and 24 hr of toxin exposure.

**Results:**

We show that toxins elicit very similar changes in the gene expression of HCT-8 cells, with the TcdB response occurring sooner. The high similarity suggests differences between toxins are due to events beyond transcription of a single cell-type and that their relative potencies during infection may depend on differential effects across cell types within the intestine. We next performed an enrichment analysis to determine biological functions associated with changes in transcription. Differentially expressed genes were associated with response to external stimuli and apoptotic mechanisms and, at 24 hr, were predominately associated with cell-cycle control and DNA replication. To validate our systems approach, we subsequently verified a novel G_1_/S and known G_2_/M cell-cycle block and increased apoptosis as predicted from our enrichment analysis.

**Conclusions:**

This study shows a successful example of a workflow deriving novel biological insight from transcriptome-wide gene expression. Importantly, we do not find any significant difference between TcdA and TcdB besides potency or kinetics. The role of each toxin in the inhibition of cell growth and proliferation, an important function of cells in the intestinal epithelium, is characterized.

## Background

*C. difficile*, a Gram-positive, spore-forming anaerobe, colonizes the human gut and causes infections leading to pseudomembranous colitis. This opportunistic pathogen flourishes in antibiotic-treated and immunocompromised patients and is frequently spread in hospitals, although community-acquired *Clostridium difficile *infection (CDI) cases have also increased [1]. The emergence of hypervirulent strains that possess more robust toxin production and increased sporulation has been correlated with outbreaks across Europe and North America [2]. In most areas, the number of cases has increased in the past decade. The number of patients hospitalized in the US with CDI doubled to approximately 250,000/year (from year 2000 to 2003) and fatalities increased at a similar rate [3]. The US healthcare costs for CDI are estimated to be over $1 billion/year [4]. As TcdA and TcdB appear to be responsible for many of the clinical manifestations of CDI, understanding the intracellular and systemic effects of each toxin is critical to developing and improving strategies for treatment and prevention.

In light of the multiple events and pathways involved in the development of CDI, we chose to examine the toxins' effects from a systems perspective, focusing on epithelial cells in vitro. Both TcdA and TcdB bind to cells [5], enter an endosome by clathrin-mediated endocytosis [6], translocate and then cleave their catalytic domain into the cytosol which glucosylates and so inactivates Rho family proteins [7]. The disruption of these crucial signaling regulators begins to explain cytotoxic effects such as deregulation of the cytoskeleton and the breakdown of the epithelial barrier [8]. However, other processes are likely affected by the trafficking and processing of these toxins. In addition, secondary effects of Rho glucosylation in relation to pathologies of CDI have not been fully elucidated.

We therefore investigated the transcriptional profile of HCT-8 [9] cells treated with TcdA or TcdB and identified pathways and cellular functions associated with differentially expressed genes. With respect to toxins, in vitro analyses of gene expression in host cells have been performed with type A botulinum neurotoxin, lethal toxin [10] and edema toxin [11] from *Bacillus anthracis*, pertussis toxin [12], Shiga toxin type 1 [13], and several others. Such studies provide lists of differentially expressed genes or classes of genes that serve as a resource for the generation of new hypotheses. In this regard, we used bioinformatics analyses to identify cellular functions altered by TcdA and TcdB that are relevant to pathogenicity. The correct identification of the majority of functions found to be affected in previous research regarding TcdA and TcdB confirmed our analysis and experimental design, and experiments reported herein validated changes in cell function that were suggested by altered gene expression.

Among the genes that TcdA and TcdB affect, many are involved in the regulation of the cell cycle and induction of apoptosis. Bacterial factors such as cytotoxic necrotizing factor and cytolethal distending toxins that disrupt normal cell cycle progression have been described as "cyclomodulins" [14]. In addition to effects of TcdA and TcdB on cells in the G_2_/M phase which have been described previously [15-18], we found that TcdA and TcdB affect expression of cyclins and cyclin-dependent kinase (CDK) inhibitors controlling the G_1_-S transition. Our experiments establish that alterations of cell cycle implicated in our analysis of gene expression do, in fact, occur in toxin-treated cells. In addition to effects on cell cycle, we also present the other cellular functions associated with differentially expressed genes, some of which enable novel hypotheses on the cellular activity and function of these toxins.

## Methods

### Cell Culture

HCT-8 cells were cultured in RPMI-1640 supplemented with 10% heat-inactivated fetal bovine serum (Gibco) and 1 mM sodium pyruvate (Gibco). The cultures were maintained at 37°C/5% CO_2 _up to passage 35. Toxin A and Toxin B, isolated from strain VPI-10643, were a generous gift from David Lyerly (TECHLAB Inc., Blacksburg, VA).

### Microarrays

HCT-8 cells (5 × 10^6^/flask) were grown overnight at 37°C/5% CO_2_. Media were replaced with 2.5 ml fresh media and toxin was added (100 ng/ml). At the end of the indicated incubation period, cells were washed with 5 ml PBS (Sigma) and total RNA was isolated using the QIAshredder and RNeasy mini kits (Qiagen), according to the manufacturer's instructions. An RNase inhibitor was added (RNasin, Promega) and samples were stored at -80°C. RNA integrity was assessed using an Agilent 2100 BioAnalyzer prior to cDNA synthesis and RNA labeling using either the 3' IVT expression or one-cycle target labeling methods (Affymetrix). Biotin-labeled RNA was hybridized to Human Genome U133 Plus 2.0 chips, washed, stained and scanned using a GeneChip System 3000 7G (Affymetrix). Data from three independent microarray experiments were deposited into the NCBI Gene Expression Omnibus repository (GSE29008).

Microarray signal intensities were normalized using the gcrma package [19]. Treatment and control groups were contrasted with linear models; a Benjamini-Hochberg correction was applied across all the probes and the nestedF method within the limma software package was used for multiple testing across all contrasts [20,21]. The Gene Ontology (GO) annotation database was used to map gene symbols to GO categories [22]. A gene symbol was considered differentially expressed if at least one of the probe sets annotated to it was significant. A probe set was considered significant if the p < 0.1 and the magnitude of the fold change was above 1.5. Enriched GO categories were identified with the topGO package using Fisher's exact test to calculate p-values and the elim algorithm [23].

### Flow Cytometry

HCT-8 cells were grown overnight to 50% confluence, media were removed and replaced with fresh media, and toxin was added at the concentrations denoted in the text and figures. At 24 h and 48 h, non-adherent cells were removed and saved on ice. Adherent cells were treated with 1 mL of 0.25% trypsin and 1 mL of Accutase with EDTA for 30 min at room temperature and joined with the non-adherent cells in 5 mL PBS. After centrifugation, resuspension for counting cells, and another round of centrifugation, the dissociated cells were resuspended to 2 × 10^6 ^cells/mL and 0.5 mL was added to 5 mL of 70% ice-cold ethanol for fixation. Afterward, the fixed cells were resuspended in 5 ml PBS with 2% Bovine Serum Albumin and then resuspended and incubated for 30 min in a solution to stain DNA (PBS with 10% Triton X-100, 2% DNasefree RNase, 0.02% propidium iodide(PI)). Single-cell fluorescence was measured with a Becton Dickinson FACSCalibur flow cytometer. The proportion of cells in each stage of the cell cycle was calculated using ModFit cell cycle analyzer. The 24 h-samples were imaged with an Amnis Imagestream imaging flow cytometer, which photographs the bright field and fluorescent channels from every cell individually [24]. Using Amnis software, a bivariate gate--based on the contrast of the brightfield image and the area of nuclear stain--differentiated apoptotic and non-apoptotic cells [25]. All other image features were taken from the Amnis software.

## Results

### Transcriptional Responses

**Figure 1:**
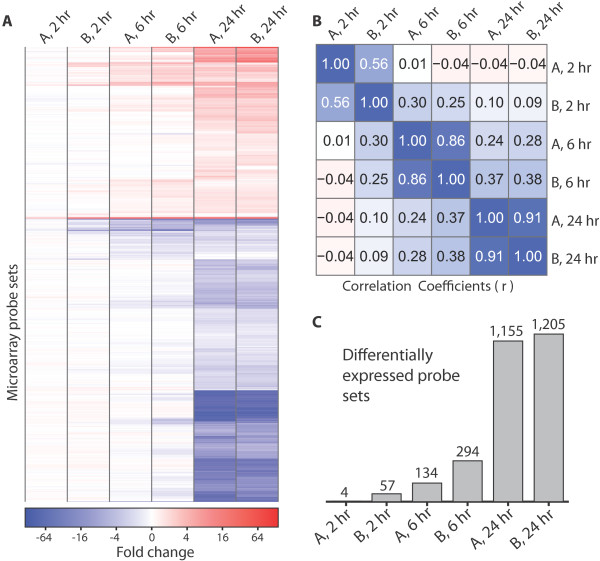
**Overall transcriptional response of HCT-8 cells to TcdA and TcdB**. A. A heatmap shows the number of differentially expressed probe sets at 2 h, 6 h, and 24 h. The color scale represents the fold change (binary log scale) of genes relative to untreated cells at the same time point. "A, 2 hr" indicates the gene expression of cells after 2 h of TcdA treatment. TcdA and TcdB concentration is 100 ng/ml. B. The correlation of transcriptional profiles between TcdA and TcdB at the indicated time points are displayed in a correlation matrix. The values represent the Pearson correlation coefficient calculated from the fold change of all the probe sets within the microarray. C. The number of differentially expressed genes used to identify enriched GO categories was determined from a linear model (Methods).

Overall, the changes in gene expression are consistent as time progresses, but the number of differentially expressed genes increases (Figure 1A). Specifically, at 2 h and 6 h, there are 4 and 134 probe sets differentially expressed (relative to control) for TcdA and 57 and 294 for TcdB, respectively (Figure 1C). Many more are differentially expressed by 24 h--1,155 and 1,205 in TcdA- and TcdB-treated cells, respectively. In order to validate these data, qRT-PCR was performed on 10 representative genes (r = 0.89 by Pearson correlation; Additional File 1; Additional File 2, Figure S1 A). Since the glucosylation of Rho family proteins occurs within one hour of toxin treatment [26], many of the differentially expressed genes at 24 h may reflect secondary effects from the initial toxin action or possibly other unknown activities and processing of the toxin.

Though the transcriptional responses to the two toxins are similar overall, a notable difference between the two toxins is that TcdB-induced changes occur more rapidly (Figure 1A). At the later time points, however, the overall transcriptional response induced by TcdA becomes more similar to the TcdB-induced transcriptional changes (see correlations in Figure 1B). Among the most affected genes, immediate early-response genes such as JUN, KLF2, and RHOB are upregulated 2 h after toxin treatment and remain increased compared to untreated cells through 24 hr (Additional File 2, Figure S1 B). While identification of the most-affected genes provides important insight, focusing on a small subset risks overlooking other toxin effects key to the disease process. We therefore analyzed the expression data in the context of broad functional categories.

### Functions associated with differentially expressed genes

**Figure 2:**
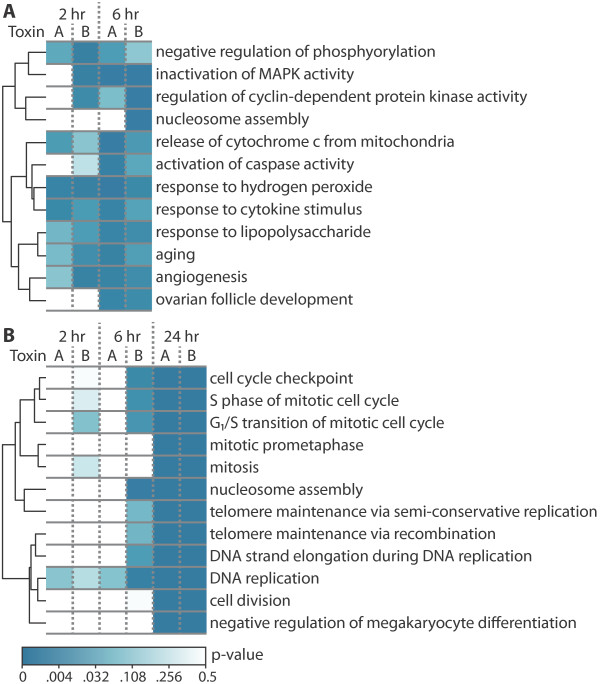
**Gene ontology categories associated with differentially expressed genes**. A. The most significantly enriched GO categories (Fisher Exact Test, topGO elim algorithm [23], see Methods) at 2 h and 6 h are displayed in a heat map. The color intensity in each cell corresponds to the p-value (Fisher Exact Test) for the GO category that is enriched. The dendrograms were generated from a hierarchical clustering of GO Groups according to Resnik similarity [49]. B. The most significantly enriched GO categories at 24 h were determined similarly.

We employed the GO database, which contains extensive annotation of biological functions associated with specific genes, to identify cellular phenotypes associated with changes in gene expression. The terms in this database are separated into three ontologies: *Molecular Functions, Cellular Components*, and *Biological Processes *(detailed descriptions at http://www.geneontology.org). A GO category--here defined as all the genes associated with a single GO term--with a proportion of differentially expressed genes greater than would be expected by chance is considered overrepresented or enriched (Methods). While some enriched categories might have been anticipated, others provide novel insights. Within the *Biological Processes *ontology, the most significantly enriched categories at 2 and 6 hr are listed in Figure 2A. Within the *Cellular Component *ontology, the *mitochondrial outer membrane *and the *apical junction complex *category are enriched most significantly at 6 h (Additional File 3, Figure S2 A). Interestingly, many of the functions related to the enriched categories have been linked with toxin treatment in previous work. One or both of the toxins induce activation of caspases [17,27-29], damage mitochondria and cause the release of cytochrome c [30,31], increase oxygen radicals and expression of cytokines [32-34], alter MAPK signaling [35-37], and disrupt the organization of tight junctions [8]. Hence, our analysis of gene expression as summarized in Figure 2 is consistent with the previously reported cellular responses to these toxins.

**Figure 3:**
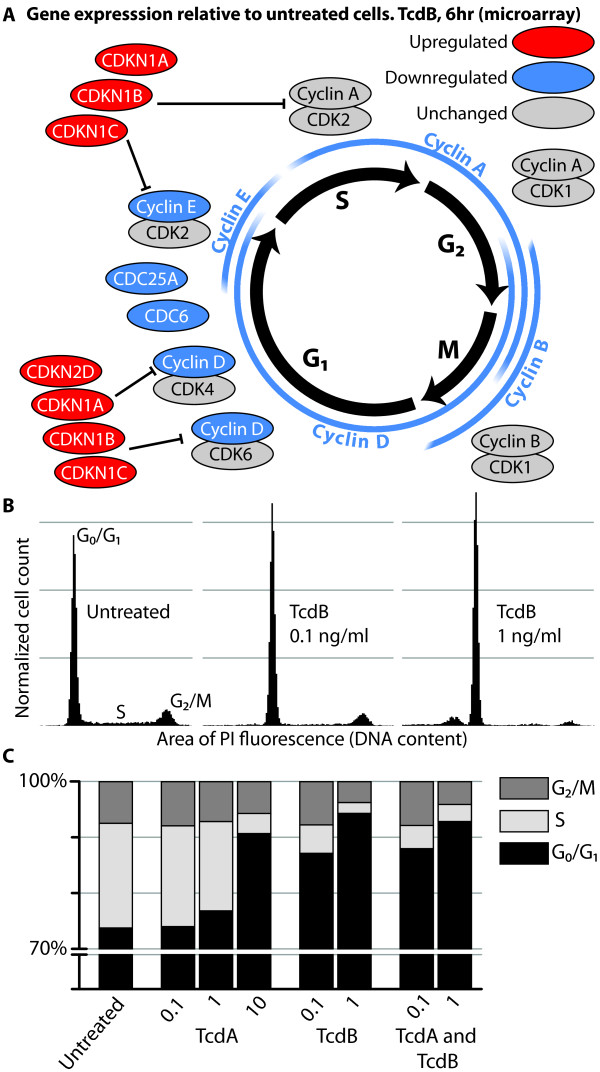
**The altered gene expression of G_1 _phase cell cycle regulators at 6 h and changes in the distribution of cells within the cell cycle**. A. A schematic of cell cycle regulation with proteins placed next to the phase of cell cycle with which they are associated (p19 and p21 are the products of the CDKN2D and CDKN1A genes, respectively). Gray, blue, and red indicate genes with unchanged, increased, or decreased expression, respectively, post toxin treatment. B. Cells in a subconfluent culture were treated with the indicated concentrations of toxin for 24 h. The DNA content of cells in each condition was quantified by PI fluorescence. The histograms of the area of PI fluorescence are normalized to the total number of cells (denoted as normalized cell count) in the sample such that the area under each histogram is equal to 1. In this way, the proportions of cells in each phase of the cell cycle may be compared for different size samples. The scale of the vertical axis is the same in each histogram. C. The percentage of cells in each phase of the cell cycle was calculated using ModFit LT software. Sub-G_0_/G_1 _cells were not included in the calculations.

The most significantly enriched categories for each toxin at the later time points are related to cell cycle and DNA replication (Figure 2B). Categories such as *telomere maintenance *and *nucleosome assembly *provide more specific connections between the toxins and changes in DNA replication. A more extensive list reveals that several categories related to microtubule organization during cell division are also enriched (Additional File 3; Figure S2 B). By 24 hr, there are changes related to virtually all elements of the cell cycle, but those controlling G_1 _and S phases are more significantly affected. Though many of the genes within the enriched categories were not among the most differentially expressed genes, the abundance of differentially expressed genes involved in the same functions provides evidence for toxin effects on control of cell cycle at the G_1 _phase. Cyclins and other proteins necessary for progression from the G_1 _phase into and through the S phase are downregulated (Figure 3A). Cyclin proteins expressed at different points are central in coordinating entry into or exit from different phases. They specifically bind and activate particular CDKs which then phosphorylate downstream targets effecting progression [38]. Inhibitors of cyclin-CDK complexes from the INK4 family (p15, p16, p18, and p19) and Cip/Kip family (p21, p27, and p57) may suppress cyclin-CDK signaling [39]. Expression of many of these and other genes, such as CDC6 and CDC25A that are required for progression from G_1 _to the S phase, is altered by TcdA and TcdB. The decreased expression of G_1 _cyclins along with the increased expression of inhibitors of G_1_-associated cyclin-CDK complexes suggest altered regulation of the cell cycle specifically in G_1_. We also measured expression of genes and proteins (Additional File 1 Additional File 4) after 6 and 24 hr of treatment with 0.1, 1, and 10 ng/ml of TcdA or TcdB in confluent and subconfluent cultures, which confirmed a consistent alteration of cell cycle genes and proteins across a variety of conditions.

### Effects of TcdA and TcdB on the Regulation of Cell Cycle

The functional changes suggested by altered gene and protein expression were then investigated by quantifying the proportion of cells in each phase of the cell cycle before and after toxin treatment. To focus on actively growing cells and avoid the effects of contact inhibition, subconfluent cultures were used. After 24 hr of 0.1 or 1 ng/ml TcdB treatment, the distribution of cells across the cell cycle changes significantly, with only a small increase in the proportion of cells with less than a G_0_/G_1 _amount of DNA--cells that are presumably dead or dying (Figure 3B). In agreement with our gene expression analysis, the percentage of G_0_/G_1 _cells increased from 67% in untreated cells to 91% and 94% in cultures treated with 10 ng/ml TcdA and 1 ng/ml TcdB, respectively (Figure 3C). The magnitude of increase in the G_0_/G_1 _proportion is also concentration-dependent. The effect on cell cycle by the combination of TcdA and TcdB is comparable to those produced by TcdB alone (Figure 3C), indicating that, with respect to their influence on cell-cycle arrest, the toxins are neither synergistic nor antagonistic. As with gene and protein expression, TcdB is more potent or faster-acting than TcdA. Taken together, these data establish that the toxin-induced alterations in genes associated with cell cycle correlate with a block at the G_1_-S interface. In other studies, a G_2_/M arrest has been reported in human cell lines treated with different concentrations of TcdA or TcdB [16-18]. This G_2_/M arrest has been linked with a deregulation of the cell structure resulting in an inability of cells to complete cytokinesis [40]. We therefore investigated the cell cycle effects and morphology of cells treated for 24 hr with higher concentrations of TcdA (100 ng/ml) and TcdB (10 and 100 ng/ml).

**Figure 4:**
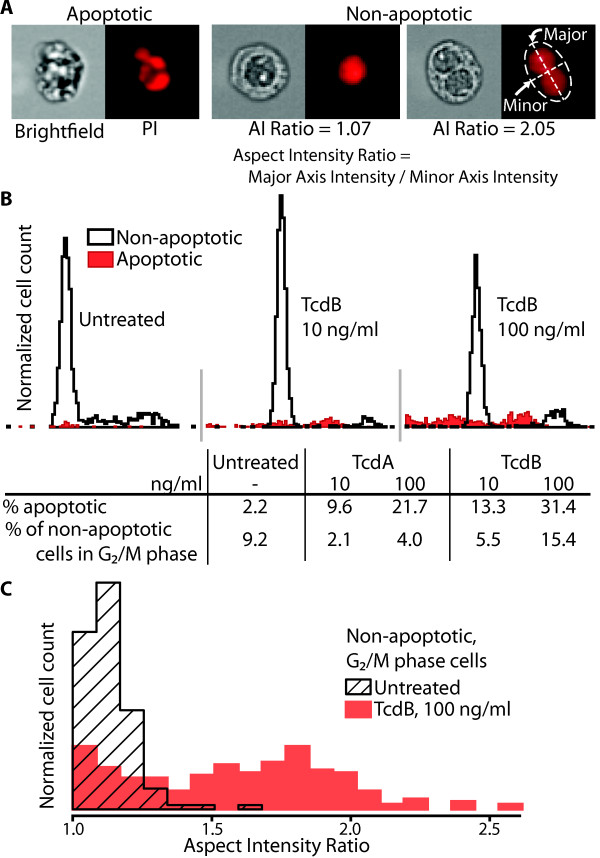
**Distribution of apoptotic versus non-apoptotic cells within the cell cycle and characteristics of G2/M phase, toxin-treated cells**. A. Cells were classified as either apoptotic or non-apoptotic based on the contrast of their brightfield image and the area of PI fluorescence. Representative images of a cell in each class are shown (100 ng/ml TcdB). B. Histograms of the area of PI fluorescence of each cell show the location of apoptotic and non-apoptotic cells within the cell cycle. The percentage of G_2_/M cells represents the proportion of non-apoptotic cells with a G_2_/M level of DNA. C. Non-apoptotic G_2_/M phase cells were analyzed to determine the number of distinct nuclei. For this analysis only, cells with an area of PI fluorescence 1.85 times greater than the PI fluorescence area at the G_0_/G_1 _peak were considered to be G_2_/M cells. The major and minor axis intensity values are the length of the axis weighted by the intensity of the image along the axis.

Our analysis of single-cell images from toxin-treated cultures reveals two unanticipated observations: (1) a biphasic distribution of apoptotic cells with respect to stage of cell cycle and (2) two populations of cells at the G_2_/M interface. Cells with a high-contrast bright-field image and a low area of PI fluorescence are classified as apoptotic (Figure 4A). Typically, apoptotic cells are associated with a PI fluorescence level less than that of the G_0_/G_1 _population. Here, a significant portion of the toxin-treated cells between the G_0_/G_1 _and G_2_/M cell populations (typically associated with/attributed to the S-phase) are apoptotic (Figure 4B). Thus, the accumulation of toxin-treated cells with S-phase levels of PI-fluorescence is not the result of progression from G_1 _but rather the apoptosis of G_2_/M cells. Even 24 hr after the addition of 100 ng/ml of TcdB, apoptosis does not dominate or override effects on cell cycle. At the highest concentration tested (100 ng/ml), 68.6% of TcdB-treated cells are still classified as non-apoptotic (Figure 4B). Of the total number of non-apoptotic cells, the proportion in the G_2_/M phase increases as the concentrations of either TcdA or TcdB increases, indicating an inhibition of progression from G_2_/M phase, in addition to the G_1_-S block discussed above.

In order to understand the differences between toxin-treated and control cells in G_2_/M, we determined several cellular characteristics (circumference, area, and others) of individual cells using an imaging flow cytometer. The feature that best distinguishes toxin-treated from untreated cells is the intensity-weighted aspect ratio of the PI fluorescence image. When an ellipse is fit around the image, an aspect ratio near one indicates a circular nucleus and a higher aspect ratio indicates an elliptical nucleus or multiple nuclei (Figure 4A). Upon visual inspection, a high aspect ratio is due typically to binucleation. The higher proportion of binucleated cells in toxin-treated cells (Figure 4C) indicates that the G_2_/M block is attributable to a failure to complete cytokinesis [40]. Therefore, in addition to demonstration of a G_1_-S block, our results show an inhibition of progression at the G_2_-M transition, which is congruent with previous findings [15-18] in other cell types treated with different toxin concentrations. Importantly, these G_2_/M effects were observed at the same concentration of toxin used for microarray analysis (100 ng/ml). Again, TcdA elicited a similar response to TcdB at the same concentration, yet to a lesser extent, thus showing consistency from gene and protein expression to cell function.

## Discussion

Understanding the differences between these two toxins is particularly relevant in determining their roles in *C. difficile *infection. Toxin A appears to be the dominant virulence factor in animal studies, yet Toxin B has higher enzymatic activity in vitro and is more potent when injected into Don cells and for human cells studied in vitro [26,41]. In a hamster model, Kuehne *et al*. found that strains of *C. difficile *producing only TcdA or TcdB are comparable in their virulence, while Lyras et al used a TcdA mutant to show that TcdB was the key virulence factor [42,43]. In this study, we used a systems approach to understand the effects of TcdA and TcdB on a human colonic epithelial cell line. We observed that the responses to these two toxins are strikingly similar, with the response to TcdB occurring more rapidly. Investigation of one of the biological consequences of these changes in gene expression revealed toxin effects at both the G_1_-S and the G_2_-M transitions.

In order to explore the interactions between *C. difficile *and intestinal epithelial cells, Janvilisri *et al*. examined the transcriptional responses of Caco-2 cells and *C. difficile *organisms during an in vitro infection [44]. Because expression was measured at no more than 2 hr post-infection, most of the changes in gene expression were slight, yet they identified functions such as cell metabolism and transport associated with affected genes. We focused on cells treated with TcdA or TcdB at a concentration and time course in which the cell morphology is strongly affected. The effects of TcdA and TcdB on gene expression in host cells have been interrogated in other studies focusing on individual pathways, but until now, an analysis of the comprehensive global transcriptional response induced by either TcdA or TcdB alone had not been performed.

Our systems approach identified a disruption of the cell cycle not readily apparent from a ranked list of genes. This approach overcame difficulties in deciphering the particular relevance of genes known to be induced by several stimuli or genes whose expression leads to many possible cellular phenotypes. JUN is overall the most differentially expressed gene in our data, and, considering TcdA or TcdB as a cellular stress, its dramatic increase in expression is consistent with it being characterized as a stress-response gene. However, increased JUN expression has also been associated with the promotion of G_1 _progression, protection from apoptosis after ultraviolet radiation, and even induction of apoptosis [45]. Clearly, multiple events may lead to the same changes in expression of an individual gene. The identification of functions associated with many of the differentially expressed genes thus provides better evidence of actual biological functions important to the toxin response.

These results have clarified the effects of TcdA and TcdB at each stage of the cell cycle. In studying Rho signaling, Welsh et al. showed that combined Rho, Rac, and Cdc42 inhibition by TcdA (200 ng/ml) in fibroblasts led to decreased cyclin D1 expression and an inability of serum-starved cells, stimulated with fetal calf serum and treated with toxin, to progress past the G_1 _phase [46]. Importantly, we show that a strong G_1 _arrest occurs in unsynchronized, proliferating epithelial cells. Only when treated with higher concentrations (100 ng/ml TcdA, 10 ng/ml TcdB) of toxin did we begin to observe the inhibition of cell division at the G_2_/M phase in a significant proportion of cells. With regard to cell death, others have shown an increased susceptibility of S-phase cells to toxin treatment [47]. We did observe an increase in the proportion of apoptotic S or G_2_/M phase cells. At low concentrations (10 ng/ml TcdA, 1 ng/ml TcdB), the decrease in the proportion of S-phase cells, however, could not be entirely accounted for by death of cells at a particular point in the growth cycle. Rather, many non-apoptotic cells remained in the G_0_/G_1 _phase.

## Conclusion

Our results have several implications in reference to the role of these toxins in pathogenicity. In a host, the gut epithelial cells normally turn over every 2-3 days [48]. Disruption of this cellular renewal process, and therefore cell cycle, impairs the maintenance of the epithelial barrier. The ability of both TcdA and TcdB to arrest growth at the G_0_/G_1 _phase and the G_2_-M transition, by likely different mechanisms (G_1 _arrest occurs even at low toxin concentrations and is associated with altered protein signaling; G_2 _arrest is likely associated with disorganization of the cytoskeleton), places each toxin in the category of cyclomodulins. As has been previously shown however, control of cell proliferation is certainly not their only or necessarily primary effect (e.g., inflammation, disruption of tight junctions). The high similarity in the gene expression induced by these two toxins indicates that, qualitatively, their effects and the overall cellular responses are comparable. The rate of internalization and/or the rapidity of inactivation of Rho-family proteins in different hosts may partially account for the different rates in the onset of gene expression. Though we did not observe synergy or antagonism between the two toxins, it is possible that each could differentially bind various cell types and therefore act synergistically within a host. Clearly, the response to each toxin is a complex process involving the activation and inhibition of several pathways in different cell types. The integration and use of the data we present here have and will continue to aid the organization of these multiple effects into a central framework for interrogating toxin activity.

## Authors' contributions

KMD performed all computational and statistical analyses, cell-cycle experiments, image processing, and drafted the manuscript. GMD performed all experiments for obtaining mRNA for microarrays and PCR. MCG, KMD, and LMC performed experiments to measure protein expression and quantify cell-cycle effects. MDS and JAL performed and contributed to the design of the flow and image cytometry experiments. All authors together conceived this study, participated in its design and coordination, and helped to draft the manuscript. All authors read and approved of the final manuscript.

## Supplementary Material

Additional file 1 **Supplementary Methods**. Materials and methods for western blotting and qRT-PCR. Click here for file

Additional file 2 **Figure S1**. qRT-PCR validation of microarray and gene expression of highly differentially expressed genes. Click here for file

Additional file 3 **Figure S2**. Enriched GO categories within the Cellular Component and Biological Process ontologies. Click here for file

Additional file 4 **Figure S3**. Gene expression from toxin-treated cells in subconfluent and confluent cultures and protein expression after treatment with various toxin concentrations. Click here for file
