# Supplementary material for: Systems analysis of the transcriptional response of human ileocecal epithelial cells to Clostridium difficile toxins and effects on cell cycle control
Source: BMC Syst Biol. 2012 Jan 6;6:2. doi: 10.1186/1752-0509-6-2 (PMC3266197; doi:10.1186/1752-0509-6-2)
Supplement: Additional file 1 — Supplementary Methods. Materials and methods for western blotting and qRT-PCR. [file 1752-0509-6-2-S1.DOCX]

**Supplementary Methods**

***Quantitative real time-PCR (qRT-PCR).*** For each gene examined, primers were designed from the target sequences retrieved from the RefSeq Sequence Database, using the Primer Express 3.0 software (Applied Biosystems). Primers were then custom made through Invitrogen Oligo Program. RNA quality control was carries out using an Agilent 2100 BioAnalyzer. Approximately 2 µg of each RNA sample was converted to cDNA. Quantitative PCRs were carried out in triplicates using equal amounts of each cDNA sample approximately equivalent to 50 ng of starting total RNA. Power SYBR Green Master Mix (Applied Biosystems, P/N 4367660) was used with the respective forward and reverse primers at the optimized concentrations. Amplifying PCR and monitoring of the fluorescent emission in real time were performed in the ABI Prism 7900HT Sequence Detection System (Applied Biosystems) as described (ABI SYBR Green Protocol). To verify that only a single PCR product was amplified per transcript, dissociation curve data was analyzed through the 7900HT Sequence Detection Software (SDS). To account for differences in starting material, quantitative PCR was also carried out for each cDNA sample using housekeeping genes synthesized in-house, human glyceraldehyde-3-phosphate dehydrogenase (GAPDH) and hypoxanthine phosphoribosyltransferase I (HPRT1). The data collected from these quantitative PCRs defined a threshold cycle number (Ct) of detection for the target or housekeeping genes in each cDNA sample. The relative quantity (RQ) of target, normalized to geometric means of the housekeeping genes and relative to a calibrator (the Rox reference dye in this case), is given by RQ = 2 ^–∆∆Ct^ where ∆∆Ct represents the difference in Ct between the transcript and the housekeeping gene for the same RNA sample. The ratio of the RQs for the treated sample and the experiment sample was used to derive the fold change.

***Western Blots.*** After toxin treatment, non-adherent cells were removed and centrifuged. The adherent cells were then treated with 200 μL of homogenization buffer: 10 mM HEPES pH 7.4, 150 mM NaCl, 10 mM sodium pyrophosphate, 10 mM NaF, 10 mM EDTA, 4mM EGTA, 0.1 mM PMSF, 2 μg/mL CLAP (Chymostatin, Leupeptin, Antipain, and Pepstatin), and 1% Triton X-100. Each well was scraped and the sample with homogenization buffer was added to the resuspended non-adherent cells. This was centrifuged at 15,000 rpm for 5 min at 4˚C and the supernatant was boiled for 5 min, placed on ice for 5 min, boiled again for 5 min, and stored at -20˚C. Samples containing equal amounts of protein were loaded into each lane of a 12.5% polyacrylamide gel. Gels were electrophoresed, transferred to PVDF, and the membranes were blocked with 5% skim milk in PBS, 0.1% Tween-20 for 2h. Primary antibodies (Cyclin D1 #2922, Cyclin E2 #4132, p57 Kip2 #2557, p27 Kip1 #2552 from Cell Signaling and Cyclin A sc-751 from Santa Cruz Biotechnology) were added to the blocking solution and membranes were incubated overnight at 4˚C on a platform shaker. The membranes were then washed six times in PBS-Tween 20. The appropriate secondary antibodies, either anti-mouse or anti-rabbit HRP-linked antibodies (#7076, #7074, Cell Signaling), were added and the membranes were incubated for 2h on a platform shaker. The membranes were subsequently washed 6 times and proteins were detected by chemiluminescence with ECL reagents (Amersham). To strip antibodies, the membranes were incubated in 50 mM Tris, 2% SDS, 0.7% β-mercaptoethanol at 50˚C for 30 min. The membranes were then washed 6 times, blocked, and reprobed with a GAPDH antibody (ab8245 from Abcam) as described above.
