# Supplementary material for: Systems analysis of the transcriptional response of human ileocecal epithelial cells to Clostridium difficile toxins and effects on cell cycle control
Source: BMC Syst Biol. 2012 Jan 6;6:2. doi: 10.1186/1752-0509-6-2 (PMC3266197; doi:10.1186/1752-0509-6-2)
Supplement: Additional file 2 — Figure S1. qRT-PCR validation of microarray and gene expression of highly differentially expressed genes. [file 1752-0509-6-2-S2.PDF]

Figure S1

A. Fold changes of gene expression relative to controls as measured by microarray were validated by PCR. To summarize multiple probe sets within a microarray that were annotated to the same gene, the value for the probe set with the greatest fold change is shown. The notation "A, 2hr" indicates the expression of genes treated with TcdA for 2 hr.

B. This list consists of genes that were among the top 25 differentially expressed genes (according to fold change) in at least 3 of the 6 conditions.

A

	A, 2 hr		B, 2h r		A, 6 hr		B, 6 hr		A, 24 hr		B, 24 hr	
	Array	PCR	Array	PCR	Array	PCR	Array	PCR	Array	PCR	Array	PCR
JUN	3.8	1.5	27.9	3.7	39.7	10.4	37.5	16.4	37.7	46.5	44.9	68.0
RHOB	2.8	1.4	8.4	3.7	18.3	11.8	21.6	12.3	14.1	16.4	13.8	23.2
CDKN1C	1.2	1.0	3.2	−1.6	3.2	3.2	6.0	11.9	3.3	2.0	1.6	1.0
CDKN1B	1.2	1.2	1.7	1.1	1.4	1.6	1.5	2.5	1.7	2.1	1.5	2.4
CCND1	1.2	1.1	−1.4	−1.3	−2.8	−2.1	−5.2	−5.4	−1.5	1.1	−1.5	1.3
CDC25A	1.3	1.7	1.1	−1.1	−1.5	−1.6	−4.2	−2.7	−11.7	−4.4	−6.2	−2.6
CCNE2	1.2	1.3	1.2	1.0	−1.4	−1.5	−4.9	−3.0	−68.6	−8.3	−13.2	−5.8
CCNA2	1.3	1.2	1.3	1.0	1.1	−1.0	1.1	1.2	−61.0	−17.9	−32	−19.4
DUSP6	−1.7	1.0	−14.7	−3.9	−12.6	−5.4	−22.1	−21.6	−4.3	−3.3	−2.5	−1.8
CTGF	1.1	−1.1	−4.5	−2.7	−28.2	−11.2	−31.9	−25.1	−98.4	−35.2	−95	−29.9

B

Symbol	Name	A, 2 hr	B, 2 hr	A, 6 hr	B, 6 hr	A, 24 hr	B, 24 hr
JUN	jun proto-oncogene	3.8	27.9	39.7	37.5	37.7	44.9
KLF2	Kruppel-like factor 2 (lung)	3.9	14.5	10.5	12.0	11.0	9.4
RHOB	ras homolog gene family, member B	2.8	8.4	18.3	21.6	14.1	13.8
DUSP6	dual specificity phosphatase 6	−1.7	−14.7	−12.6	−22.1	−4.3	−2.5
EPHA2	EPH receptor A2	−1.1	−5.3	−8.4	−15.4	−5.4	−2.3
TNFSF15	tumor necrosis factor (ligand) superfamily, member 15	−1.5	−4.0	−6.2	−9.7	−2.8	−1.5
CTGF	connective tissue growth factor	1.1	−4.5	−28.2	−31.9	−98.4	−95.0
ERG1	early growth response 1	−1.5	−4.0	−11.5	−23.4	−8.9	−22.2
AMOTL2	angiomotin like 2	−1.3	−7.5	−12.9	−13.0	−19.5	−19.9
CYR61	cystein-rich angiogenic inducer, 61	−1.9	−8.1	−8.7	−10.9	−25.8	−23.7
