# Supplementary material for: Systems analysis of the transcriptional response of human ileocecal epithelial cells to Clostridium difficile toxins and effects on cell cycle control
Source: BMC Syst Biol. 2012 Jan 6;6:2. doi: 10.1186/1752-0509-6-2 (PMC3266197; doi:10.1186/1752-0509-6-2)

Figure S2

A. Cellular Component GO categories with $p<10^{-3}$ across all time points are shown. Criteria for calculating p values and GO categories were the same as in Figure 2.

B. The 25 most significant GO Biological Processes at 24 hr were selected by the criteria described in Figure 2.

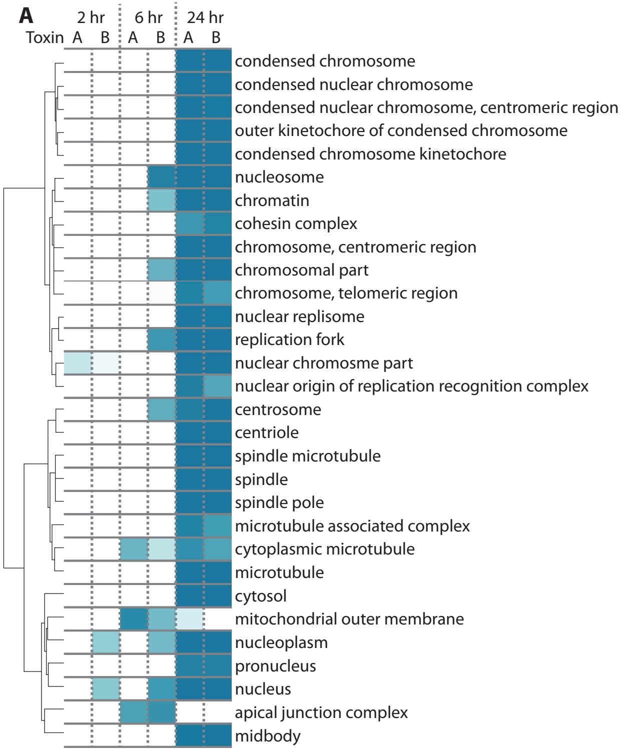

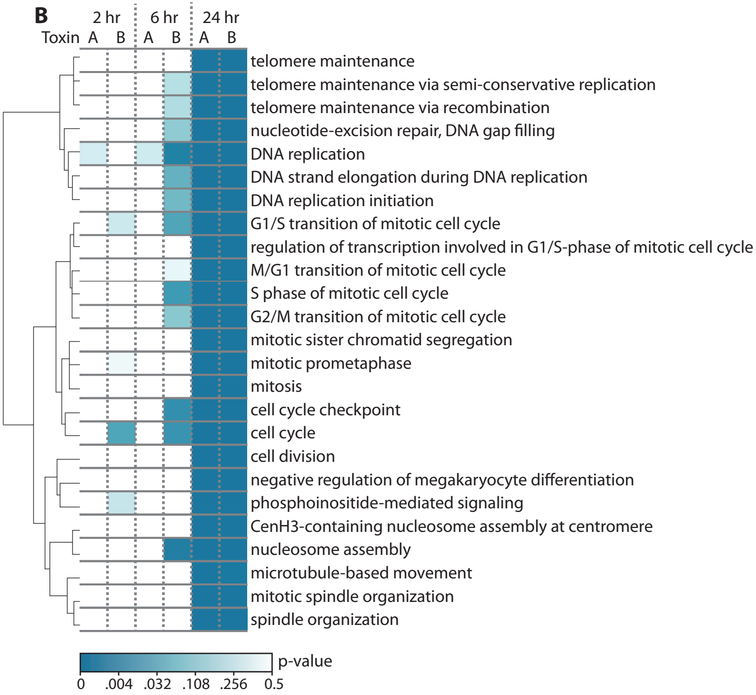

Supplement: Additional file 3 — Figure S2. Enriched GO categories within the Cellular Component and Biological Process ontologies. [file 1752-0509-6-2-S3.PDF]
