# Supplementary material for: Systems analysis of the transcriptional response of human ileocecal epithelial cells to Clostridium difficile toxins and effects on cell cycle control
Source: BMC Syst Biol. 2012 Jan 6;6:2. doi: 10.1186/1752-0509-6-2 (PMC3266197; doi:10.1186/1752-0509-6-2)

Figure S3

A. The expression of 10 genes in both subconfluent and confluent toxin-treated cells was measured by qRT-PCR. Fold changes shown are relative to untreated samples.

B. Densitometry was performed on immunoblots of lysates from toxin-treated, confluent cells. The intensity of each band was normalized to the intensity of GAPDH, the loading control. The values above each row indicate the amount of protein relative to the amount in untreated cells. The relative intensity for p57 was not calculated because the protein was not detectable in untreated cells. The blots show the presence of p57 after toxin treatment.

C. A Rac1 antibody (BD#610650) that recognizes non-glucosylated Rac1 (protein that has not been glucosylated by either toxin) shows the activity of TcdA and TcdB in HCT-8 cells.

A

	**A, 6 hr**		**B, 6 hr**		**A, 24 hr**		**B, 24 hr**		
Confluence	High	Low	High	Low	High	Low	High	Low	ng/ml
CCND1	1.1	1.6	−2.9	−4.2	−1.5	−2.1	−1.4	−1.4	10
	1.0	1.8	−1.1	−1.9	1.0	−1.3	−1.4	−1.8	1
	1.2	1.5	1.0	1.1	1.1	−1.3	1.0	−1.9	0.1
CCNE2	−1.1	3.9	−1.9	1.4	−1.9	−3.7	−6.8	−3.1	10
	1.0	4.0	−1.1	2.4	1.0	−1.3	−2.1	−3.6	1
	1.0	3.8	−1.1	2.8	1.1	−1.2	−1.1	−2.2	0.1
CCNA2	1.0	4.3	−1.1	4.3	−1.4	−3.3	−11.7	−8.9	10
	1.0	4.0	−1.1	4.3	1.0	−1.1	−1.5	−13.4	1
	1.1	3.6	1.0	4.2	−1.1	1.2	1.0	−1.4	0.1
CDKN1C	−0.5	5.1	3.2	51.6	2.3	3.2	3.8	1.9	10
	1.0	3.8	−1.1	18.1	1.2	1.3	2.5	4.1	1
	−1.4	3.5	−1.1	4.7	1.3	1.0	1.0	2.4	0.1
CDKN1B	1.1	4.5	1.8	4.9	1.1	1.8	2.0	2.8	10
	1.0	4.1	1.3	4.6	1.0	1.3	1.2	2.6	1
	1.1	4.3	1.1	3.1	−1.1	−1.1	−1.2	1.4	0.1
CDC25A	1.2	1.7	−1.7	−1.2	−2.0	−3.6	−6.1	−4.9	10
	1.2	1.7	1.2	1.3	1.0	−1.1	−2.2	−7.9	1
	1.3	1.7	1.2	2.4	1.0	1.1	−1.1	−1.5	0.1
DUSP6	−1.2	2.9	−6.7	−4.6	1.1	−2.3	−2.3	−1.2	10
	1.0	4.0	−1.4	−1.5	1.0	1.1	−1.4	−1.76	1
	1.1	4.1	−1.1	3.3	−1.1	−1.1	1.0	−1.4	0.1
JUN	1.5	6.1	4.3	11.0	8.9	14.3	26.1	37.1	10
	−1.1	4.2	1.8	9.6	2.0	2.9	11.8	34.4	1
	−1.3	3.5	−1.2	3.7	1.2	−1.1	2.4	7.9	0.1
RHOB	2.1	12.3	8.1	34.2	6.3	10.2	15.9	19.1	10
	1.2	5.4	3.3	29.3	1.7	3.1	8.1	16.5	1
	1.0	4.1	1.5	6.0	1.0	−1.1	2.1	6.0	0.1
CTGF	−1.6	2.7	−31.2	−11.1	−3.5	−22.7	−38.1	−20.1	10
	1.1	2.0	−1.34	−4.0	1.0	−1.3	−5.4	−30.9	1
	−1.3	2.2	−1.34	1.9	−1.0	−1.1	−1.1	−7.1	0.1

B

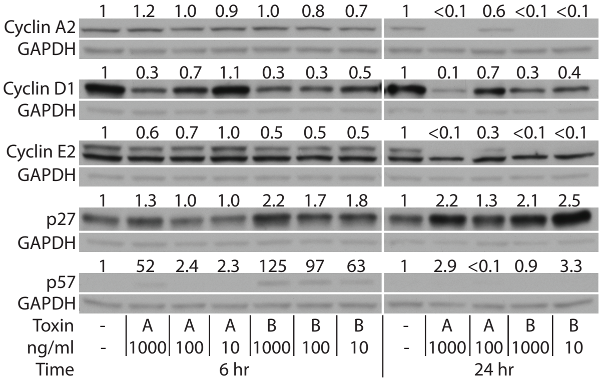

C

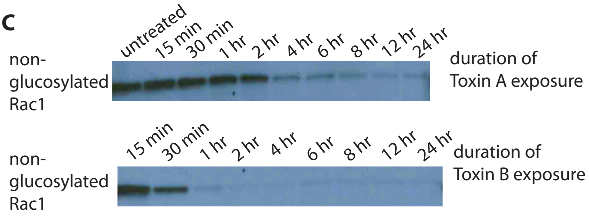

Supplement: Additional file 4 — Figure S3. Gene expression from toxin-treated cells in subconfluent and confluent cultures and protein expression after treatment with various toxin concentrations. [file 1752-0509-6-2-S4.PDF]
